# PEMFs Restore Mitochondrial and CREB/BDNF Signaling in Oxidatively Stressed PC12 Cells Targeting Neurodegeneration

**DOI:** 10.3390/ijms26136495

**Published:** 2025-07-05

**Authors:** Stefania Merighi, Mercedes Fernandez, Manuela Nigro, Alessia Travagli, Filippo Caldon, Simona Salati, Pier Andrea Borea, Ruggero Cadossi, Katia Varani, Stefania Gessi

**Affiliations:** 1 Department of Translational Medicine, University of Ferrara, 44121 Ferrara, Italy; mercedes.fernandez@unife.it (M.F.); manuela.nigro@unife.it (M.N.); alessia.travagli@unife.it (A.T.); filippo.caldon@unife.it (F.C.); vrk@unife.it (K.V.); 2 Igea Biophysics Laboratory, 41012 Carpi, Italyr.cadossi@igeamedical.com (R.C.); 3 University of Ferrara, 44121 Ferrara, Italy; bpa@unife.it

**Keywords:** Alzheimer’s disease, PEMFs, neuroprotection, PC12 cells, oxidative stress, MMP, ERK, cAMP, CREB, BDNF

## Abstract

Alzheimer’s disease (AD), the most prevalent form of neurodegenerative dementia, is characterized by progressive cognitive decline and neuronal loss. Despite advances in pharmacological treatments, current therapies remain limited in efficacy and often induce adverse effects. Increasing evidence highlights oxidative stress, mitochondrial dysfunction, and disrupted neurotrophic signaling as key contributors to AD pathogenesis. Pulsed electromagnetic fields (PEMFs) are emerging as a non-invasive, multifactorial approach with promising biological effects. In this study, we investigated the neuroprotective potential of PEMFs in NGF-differentiated PC12 cells exposed to hydrogen peroxide (H_2_O_2_) or amyloid-β peptide (Aβ), both of which model pathological features of AD. PEMF treatment significantly counteracted H_2_O_2_- and Aβ-induced cytotoxicity by restoring cell viability, reducing reactive oxygen species production, and improving catalase activity. Furthermore, PEMFs preserved the mitochondrial membrane potential and decreased caspase-3 activation and chromatin condensation. Mechanistically, PEMFs inhibited ERK phosphorylation and enhanced cAMP levels, CREB phosphorylation, and BDNF expression, pathways known to support neuronal survival and plasticity. In conclusion, these findings suggest that PEMFs modulate multiple stress response systems, promoting neuroprotection under oxidative and amyloidogenic conditions.

## 1. Introduction

Alzheimer’s disease (AD), the predominant form of dementia, represents an urgent health challenge worldwide. With the increase in life expectancy, the incidence of AD is generally increasing as well, and it is estimated to be doubled by 2050. AD is a progressive neurodegenerative disorder characterized by initial memory impairment and cognitive decline. The causes are not yet well known; nevertheless, aging, genetics, and the environment seem to be the factors involved [1]. AD is marked by memory loss and cognitive decline that become worse over time. This is caused by neuropathological features like β-amyloid (Aβ) plaques, neurofibrillary tangles (NFTs), astrogliosis, and neuronal loss, which are all closely linked to oxidative stress, mitochondrial dysfunction, and regulated cell death pathways [2,3,4,5,6,7]. Reactive oxygen species (ROS) are produced mostly by altered mitochondria; they increase Aβ and tau accumulation and cause neuroinflammation and cell death. These changes disrupt neuronal homeostasis and contribute to synaptic failure and cognitive decline. AD causes the dysregulation of important signaling proteins that are essential in neuronal survival and plasticity, including CREB and ERK. Oxidative stress caused by Aβ may inhibit the transcription of neuroprotective genes such as BDNF through CREB, and abnormal ERK activity has been linked to inflammation and tau phosphorylation [8,9]. Current FDA-approved treatments, such as memantine, acetylcholinesterase inhibitors, and anti-amyloid antibodies, may alleviate symptoms or target disease-modifying pathways, but their long-term effectiveness is still unknown, and their clinical advantages are still restricted [10]. Pulsed electromagnetic fields (PEMFs) are non-invasive biophysical stimuli that can alter cellular function. PEMFs can influence the cell membrane and its embedded molecules, including voltage-gated calcium channels, due to the very high gradient of the electric field at this location, affecting differentiation and proliferation. In addition, they reduce oxidative stress, improve mitochondrial function, and enhance synaptic plasticity [11]. Regarding their effects on neurons, data suggest that they can control molecular pathways such as PI3K/Akt, CREB, and MAPK/ERK, which supports the differentiation and survival of human bone marrow-derived mesenchymal stem cells [12]. PEMFs have shown anti-inflammatory and anti-apoptotic properties, along with increased neuronal survival, in both in vitro and in vivo models of neurodegeneration. Through a number of molecular pathways, PEMFs may specifically have beneficial impacts by regulating cell viability, proliferation, apoptosis, and necrosis, which may have important neuroprotective effects at the neural level [13]. PEMFs support neuronal survival against hypoxia by reducing ROS production, increasing antioxidant enzyme activity, and activating protective signaling cascades like the p38 MAPK pathway and CREB, according to experimental studies conducted using neuronal cell lines like PC12 and SH-SY5Y [14,15,16,17]. Furthermore, PEMFs support neuronal regeneration and plasticity by promoting the production of neurotrophic and anti-apoptotic proteins such as BDNF and Bcl-2 [17,18]. In terms of synaptic plasticity, PEMFs have been shown to influence neurite outgrowth and enhance intracellular cAMP levels, critical mediators of synaptic remodeling in MN9D dopaminergic neurons [19]. Additionally, PEMFs may modulate gene expression in peripheral blood mononuclear cells, potentially rebalancing dysregulated pathways in AD, and stimulate epigenetic regulation via miRNAs, which could aid in disease management, although the underlying mechanisms remain to be fully elucidated and require further validation [20]. Recently, in SHSY5Y and N9 cells insulted with hydrogen peroxide (H_2_O_2_) and with an analog of peptide Aβ_1-42_, O-acyl isopeptide (CP), PEMFs reduced ROS, improved mitochondrial function, and reduced cell death, providing substantial evidence of their efficacy in mitigating neuronal damage [15]. In this context, the present study aims to explore the potential neuroprotective effects of PEMFs in a PC12 cell model subjected to Aβ- and H_2_O_2_-induced injury. By assessing cell viability, mitochondrial function, oxidative stress, and the activation of relevant signaling pathways, this in vitro investigation seeks to contribute to the understanding of PEMF-mediated mechanisms of neuronal resilience.

## 2. Results

### 2.1. PC12 Morphology

NGF-differentiated PC12 cells injured with H_2_O_2_ and CP have been used in this study as a cellular model of AD to investigate the effects of PEMFs. When maintained in standard medium, the majority of PC12 cells show an irregularly polyhedral shape, with some exhibiting thin and short membrane protrusions (Supplementary Figure S1A). Under exposure to NGF, cells differentiate, showing neurite-like processes (Supplementary Figure S1B). The treatment of NGF-differentiated PC12 cells with 1 mM H_2_O_2_ induces changes in cell morphology, and cells lose their ramifications and acquire a round shape (Supplementary Figure S1C). When differentiated cells are treated with 20 μM CP, they change from having a flat shape with extensions to a swollen shape (Supplementary Figure S1D).

### 2.2. PEMFs’ Effects on Viability of PC12 Cells

**Figure 1:**
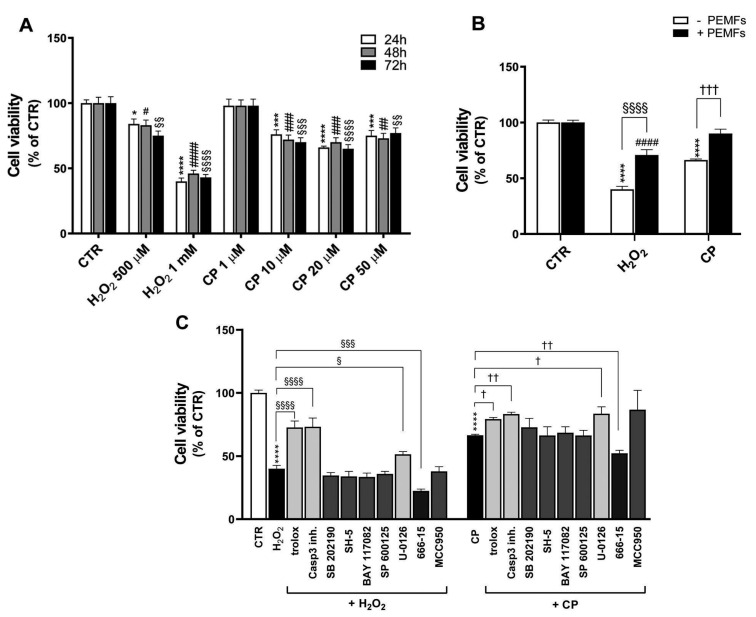
H_2_O_2_- and CP-induced cytotoxicity in PC12 cells. (**A**) Cells were treated with different concentrations of H_2_O_2_ and CP for 24, 48, and 72 h. Results are presented as mean ± SEM values of at least three independent experiments performed in duplicate (* *p* < 0.05, *** *p* < 0.001, **** *p* < 0.0001 vs. control (CTR) for 24 h; ^#^ *p* < 0.05, ^##^ *p* < 0.01, ^###^ *p* < 0.001, ^####^ *p* < 0.0001 vs. CTR for 48 h; ^§§^ *p* < 0.01, ^§§§^ *p* < 0.001, ^§§§§^ *p* < 0.0001 vs. CTR for 72 h). (**B**) Cell viability was studied in 1 mM H_2_O_2_- and 20 μM CP-injured cells for 24 h with and without PEMFs. Results are presented as mean ± SEM values of at least seven independent experiments performed in duplicate (**** *p* < 0.0001 vs. CTR without PEMFs; ^####^ *p* < 0.0001 vs. CTR with PEMFs; ^§§§§^ *p* < 0.0001, ^†††^ *p* < 0.001). (**C**) Effects of cell pretreatment for 30 min with 500 μM Trolox, 1 μM caspase-3 inhibitor, SB 202190, SH-5, BAY 117082, SP 600125, U-0126, MCC950, and 10 μM 666-15 on the viability of PC12 cells injured with 1 mM H_2_O_2_ or 20 μM CP. Results are presented as mean ± SEM values of at least three independent experiments performed in duplicate (**** *p* < 0.0001 vs. CTR; ^§§§§^ *p* < 0.0001, ^§§§^ *p* < 0.001, ^§^ *p* < 0.05; ^††^ *p* < 0.01, ^†^ *p* < 0.05). Statistical analysis was performed by one-way analysis of variance (ANOVA) and Sidak’s multiple comparison test. PEMFs, pulsed electromagnetic fields.

Two different molecules were used as stimuli to induce cell death: H_2_O_2_ at 500 μM and 1 mM and CP at different concentrations ranging from 1 to 50 μM. They were applied to the cells for 24, 48, and 72 h; then, the MTS viability assay was performed. The results included in Figure 1A show that both H_2_O_2_ (500 μM and 1 mM) and CP (10, 20, and 50 μM) at all times investigated significantly impaired neuronal viability. Based on these results, the two injury conditions chosen for subsequent analyses were 1 mM H_2_O_2_ and 20 μM CP, both after 24 h of treatment. Under these experimental conditions, cell viability was significantly decreased compared with control cells (CTR versus H_2_O_2_ and CP, 100 ± 3 versus 40 ± 3 and 66 ± 1%, respectively, **** *p* < 0.0001). Interestingly, as shown in Figure 1B, PEMFs applied for 24 h continuously were able to partially revert cell death induced by H_2_O_2_ and CP (71 ± 5%, ^§§§§^ *p* < 0.0001 and 90 ± 4%, ^†††^ *p* < 0.001, respectively), indicating a neuroprotective effect of PEMFs against both toxic stimuli. We first investigated the oxidative stress pathway by treating the cells with 500 μM Trolox for 30 min before exposing them to the insults, in order to explore possible mechanisms that could explain the compromised cell viability observed. Trolox is a vitamin E analog with antioxidant properties. As observed in Figure 1C, Trolox counteracted cell death in 1 mM H_2_O_2_-treated cells (73 ± 5% versus 40 ± 3%, ^§§§§^ *p* < 0.0001) and in 20 μM CP-treated cells (79 ± 1% versus 66 ± 1%, ^†^ *p* < 0.05), supporting the hypothesis that oxidative stress is an important contributor to the cell death induced by these treatments. We then explored programmed cell death via the inhibition of the pathway mediated by caspase-3, one of the most studied due to its pivotal role in apoptosis. For this purpose, cells were preincubated with a caspase-3 inhibitor (1 μM, 30 min) before adding the injury stimuli. The caspase-3 inhibitor induced a significant increase in the percentage of viable cells in the H_2_O_2_-treated group (73 ± 7% versus 40 ± 3%, ^§§§§^ *p* < 0.0001) and in the CP-treated group of cells (83 ± 1% versus 66 ± 1%, ^††^ *p* < 0.01) (Figure 1C). In order to investigate possible molecular mechanisms to explain the effects of PEMFs in attenuating H_2_O_2_- and CP-induced cytotoxicity and apoptosis in PC12, we screened inhibitors of different proteins and transcription factors known to be involved in pro- or anti-survival cellular pathways through the MTS assay. In particular, we tested 1 μM SB 202190 (P38 inhibitor), 1 μM SH-5 (Akt inhibitor), 1 μM BAY 117082 (NFkB inhibitor), 1 μM SP 600125 (JNK inhibitor), 1 μM U-0126 (MEK ERK1/2 inhibitor), 1 μM MCC950 (NLRP3 inhibitor), and 10 μM 666-15 (CREB inhibitor). Cells were preincubated with inhibitors for 30 min before being treated with injury stimuli, H_2_O_2_ and CP, for 24 h. The inhibitors alone did not exert any cytotoxic effects. Among all molecules tested, U-0126 and 666-15 were found to be involved in cell death induced by both H_2_O_2_ and CP (Figure 1C). U-0126 in part rescued cells from death caused by H_2_O_2_ (52 ± 2% versus 40 ± 3%, ^§^ *p* < 0.05) and CP (83 ± 6% versus 66 ± 1%, ^††^ *p* < 0.01). Molecule 666-15 worsened cell death induced by both H_2_O_2_ (22 ± 2% versus 40 ± 3%, ^§§§^ *p* < 0.001) and CP (52 ± 2% versus 66 ± 1%, ^†^ *p* < 0.05, respectively), suggesting a protective role for CREB signaling in this model.

### 2.3. PEMFs’ Effects on Cleaved Caspase-3 Expression and Chromatin Condensation in PC12 Cells

**Figure 2:**
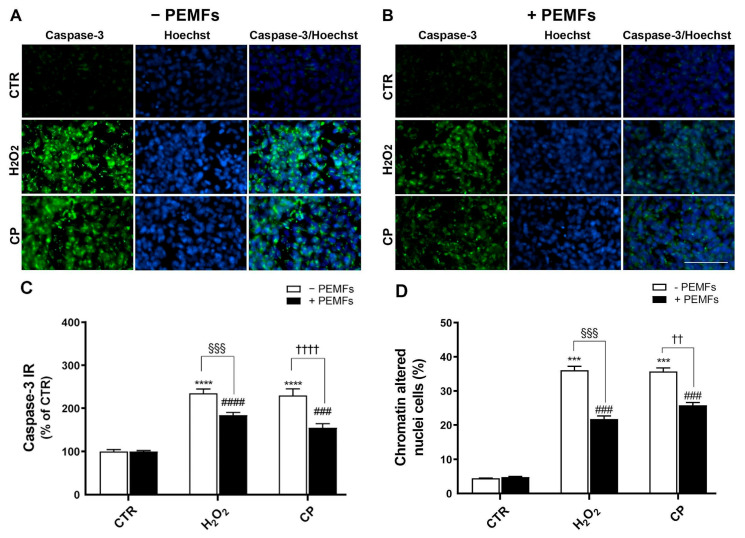
PEMFs’ effects on cleaved caspase-3 in PC12 cells. Representative images of cleaved caspase-3-positive PC12 cells (green) in control (CTR) group and with 1 mM H_2_O_2_ or 20 μM CP treatment for 90 min, without (**A**) and with (**B**) PEMFs. Hoechst 33342 nuclear staining (blue) and a merged image of cleaved caspase-3/Hoechst 33342 nuclear staining are included. Scale bar 100 μm. (**C**) Analysis of cleaved caspase-3 immunoreactivity (IR) normalized to the number of cells. Results are presented as mean ± SEM values of at least three independent experiments performed in duplicate (**** *p* < 0.0001 vs. CTR without PEMFs; ^####^ *p* < 0.0001 and ^###^ *p* < 0.001 vs. CTR with PEMFs, respectively; ^§§§^ *p* < 0.001; ^††††^ *p* < 0.0001). (**D**) Analysis of Hoechst 33342 chromatin-altered nuclei in cells. Data are presented as mean ± SEM values (*** *p* < 0.001 vs. CTR without PEMFs; ^###^ *p* < 0.001 vs. CTR with PEMFs; ^§§§^ *p* < 0.001; ^††^ *p* < 0.01). Statistical analysis was performed by one-way ANOVA and Sidak’s multiple comparison test.

In order to explore the ability of PEMFs to rescue cell viability in the H_2_O_2_- and CP-treated cells, and since caspase-3 seems to be involved in cell death, we studied caspase-3 protein expression by immunocytochemistry. The treatment of cells with H_2_O_2_ and CP for 90 min induced a significant increase in the expression of cleaved caspase-3 (235 ± 10% and 230 ± 15% versus 100 ± 4% **** *p* < 0.0001, respectively), indicating the activation of apoptotic pathways in response to both oxidative and amyloidogenic injury. This increase was notably counteracted by continuous exposure to PEMFs for 90 min, applied immediately after the treatments with H_2_O_2_ and CP, which reduced the cleaved caspase-3 levels to 184 ± 6% (H_2_O_2_-treated cells, ^§§§^ *p* < 0.001) and 155 ± 9% (CP-treated cells, ^††††^ *p* < 0.0001), suggesting a partial protective effect of PEMFs against apoptosis. Representative images of cleaved caspase-3 and Hoechst 33342 double-stained cells are included in Figure 2A,B. The results obtained from the analysis of the cleaved caspase-3 fluorescent intensity normalized to the total number of cells, expressed as a percentage of the control group, are included in the graph (Figure 2C).

The analysis of Hoechst 33342 nuclear staining shows that the treatments with H_2_O_2_ and CP for 90 min induced an increase in the percentage of cells showing chromatin-altered nuclei (36 ± 2% and 36 ± 1% versus 4 ± 1%, respectively, *** *p* < 0.001). Exposure to PEMFs significantly reduced this percentage to 22 ± 1% in H_2_O_2_-treated cells (^###^ *p* < 0.001) and 26 ± 1% in CP-treated cells (^§§§^ *p* < 0.001; ^††^ *p* < 0.01), confirming a protective role also at the nuclear level (Figure 2D). No significant differences were observed between the PEMF-exposed and non-exposed control groups (4 ± 1% versus 5 ± 1%), indicating that PEMFs do not affect the nuclear morphology under basal conditions. To further support these findings, we analyzed the mean fluorescence intensity of Hoechst 33342-stained nuclei across all experimental groups. The results mirrored those obtained from the quantification of chromatin-condensed nuclei (Supplementary Figure S2), reinforcing the association between increased Hoechst 33342 fluorescence and chromatin condensation, a hallmark of apoptotic cells [21,22].

### 2.4. PEMFs’ Effects on Oxidative Stress and Catalase (CAT) Activity in PC12 Cells

**Figure 3:**
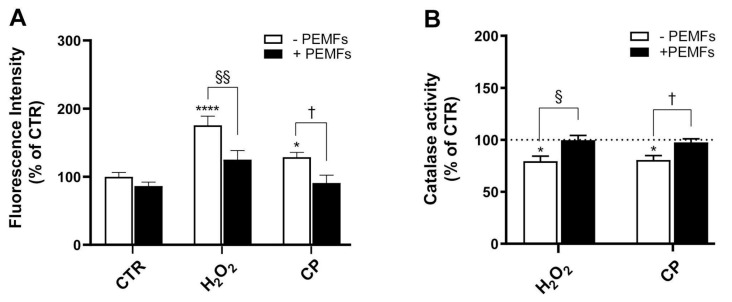
PEMFs’ effects on oxidative stress and catalase enzyme activity in H_2_O_2_- and CP-injured PC12 cells. (**A**) ROS levels in PC12 cells treated with 1 mM H_2_O_2_ or 20 μM CP for 24 h through H_2_DCFDA test. Results are presented as mean ± SEM values of at least five independent experiments performed in duplicate (**** *p* < 0.0001 and * *p* < 0.05 vs. control (CTR) without PEMFs, respectively; ^§§^ *p* < 0.01; ^†^ *p* < 0.05). (**B**) Catalase activity measured after cell treatment with 200 μM H_2_O_2_ or 20 μM CP for 4 h. Results are presented as mean ± SEM values of at least four independent experiments performed in duplicate (* *p* < 0.05 vs. CTR without PEMFs; ^§^ *p* < 0.05; ^†^ *p* < 0.05). Statistical analysis was performed by one-way ANOVA and Sidak’s multiple comparison test. ROS, reactive oxygen species; H_2_DCFDA, 2′,7′-dichlorodihydrofluorescein diacetate.

The fluorogenic dye H_2_DCFDA measures ROS activity within the cell. The fluorescent intensity measured is proportional to the ROS levels within the cell cytosol, which are known to induce DNA damage and oxidize proteins and lipids, consequently causing oxidative stress. We investigated the ROS levels after 24 h of treatment with both 1 mM H_2_O_2_ and 20 μM CP. As can be observed in Figure 3A, H_2_O_2_ and CP significantly increased the percentage of fluorescent intensity due to ROS production compared to untreated control cells (176 ± 13% and 129 ± 7% versus 100 ± 6%, **** *p* < 0.0001, * *p* < 0.05, respectively), confirming that both toxic stimuli promote oxidative stress in this model. Interestingly, exposure to PEMFs for 24 h immediately after the treatment with H_2_O_2_ and CP significantly attenuated ROS production (125 ± 13% and 91 ± 11%, ^§§^ *p* < 0.01 and ^†^ *p* < 0.05, respectively) (Figure 3A).

Given these results, we next examined whether PEMF exposure could also modulate the activity of endogenous antioxidant defense systems, focusing on CAT, an enzyme that catalyzes the dismutation of H_2_O_2_ into O_2_ and H_2_O. In particular, PC12 cells were treated with 200 μM H_2_O_2_ and 20 μM CP for 4 h, with or without PEMF exposure, and then the CAT activity was assessed. As shown in Figure 3B, the treatment with H_2_O_2_ and CP induced a decrease in CAT activity compared to the control group (82 ± 6% and 85 ± 3% versus 100 ± 3%, * *p* < 0.05). The exposure of cells to PEMFs significantly reverted the CAT activity to near control levels after treatment with H_2_O_2_ and CP (100 ± 5% and 100 ± 4%, ^§^ *p* < 0.05 and ^†^ *p* < 0.05, respectively) (Figure 3B). These data support the hypothesis that PEMFs exert a protective antioxidant effect, not only by reducing ROS accumulation but also by preserving the activity of the CAT enzyme.

### 2.5. PEMFs’ Effects on MMP in PC12 Cells

**Figure 4:**
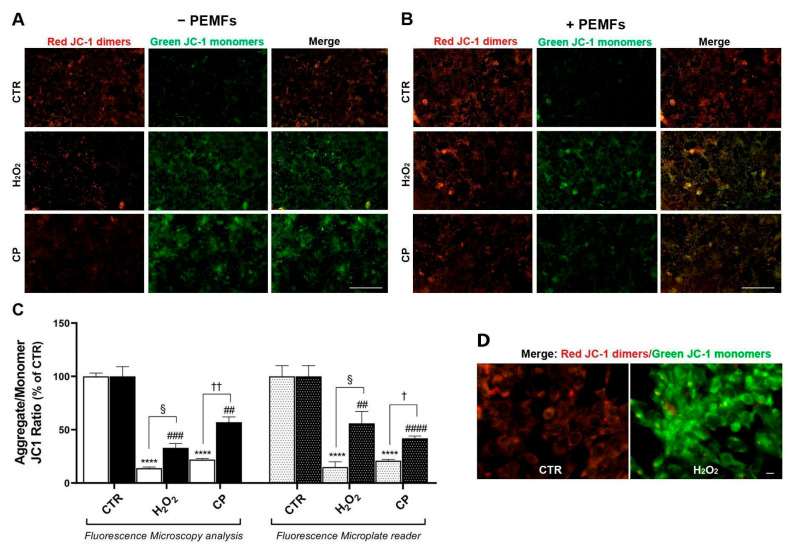
PEMFs’ effects on MMP depolarization in PC12 cells. Representative images of JC-1 staining in 1 mM H_2_O_2_- or 20 μM CP-treated cells for 90 min, without (**A**) and with PEMFs (**B**). Scale bar 100 μm. (**C**) The graph includes the results of fluorescence microscope images and fluorescence microplate reader analyses, expressed as the red/green ratio percentage of the control group (CTR). These results are presented as mean ± SEM values of at least three independent experiments performed in duplicate (**** *p* < 0.0001 vs. CTR without PEMFs; ^###^ *p* < 0.001, ^##^ *p* < 0.01, ^#####^ *p* < 0.0001 vs. CTR with PEMFs; ^§^ *p* < 0.05; ^†^ *p* < 0.05, ^††^ *p* < 0.01). Statistical analysis was performed by one-way ANOVA and Sidak’s multiple comparison test. (**D**) Representative high-magnification images of JC-1-stained cells under CTR and after treatment with 1 mM H_2_O_2_. Scale bar 10 μm. MMP, mitochondrial membrane potential.

For the study of MMP, we used JC-1, a cationic carbocyanine dye (green) that accumulates in mitochondria in a potential-dependent manner. In control cells, JC-1 enters the mitochondria, showing red fluorescent aggregates (dimers). By contrast, in injured cells, due to the decrease in MMP, JC-1 enters the mitochondria but in lower amounts, which are not sufficient to form aggregates, thus maintaining its original green fluorescence (monomers). Cells were loaded with JC-1 and subjected to injury stimuli—1 mM H_2_O_2_ or 20 μM CP—for 90 min in both conditions, with or without PEMF exposure. We used two different technical approaches for the MMP study: live microscopy imaging and fluorescence plate reading. For the live microscopy imaging, cells were grown in eight-well chamber slides and processed as described. At the end of the treatments, cells were immediately analyzed under fluorescence microscopy. Representative images are included in Figure 4, where dimers (red) and monomers (green) can be identified in the control and injury-treated groups, respectively. As can be observed, both stimuli, H_2_O_2_ and CP, induced a significant increase in green monomers (Figure 4A), indicating MMP depolarization, whereas, in the PEMF-exposed group, the stimuli’s effects were lower and dimer/monomer colocalization could be observed (Figure 4B). The results obtained from the analysis of the mean fluorescent intensity expressed as a red/green ratio percentage of the control group (relative ratio) are included in the graph in Figure 4C. Treatment with 1 mM H_2_O_2_ and 20 μM CP induced a significant decrease in the red/green ratio % (14 ± 1% and 22 ± 1% versus 100 ± 3%, **** *p* < 0.0001), and PEMF exposure in part reverted this effect (33 ± 4% and 57 ± 5% versus 100 ± 9%, ^###^ *p* < 0.001 and ^##^ *p* < 0.01, respectively; ^§^ *p* < 0.05 and ^††^ *p* < 0.01). The staining of JC-1 at higher magnification can be observed as red dimers or green monomers in control and H_2_O_2_-treated cells, respectively (Figure 4D). For fluorescence-based measurements, cells were seeded in black 96-well microplates to minimize background signals and optimize the detection sensitivity. At the end of the treatments, fluorescence was measured using a multimode plate reader. The fluorescence intensity values, corresponding to aggregate and monomer species, were analyzed and expressed as the JC-1 ratio aggregate/monomer as a percentage of the control group. The results confirmed the trend observed from the live cell imaging in the MMP analysis. Specifically, treatment with 1 mM H_2_O_2_ and 20 μM CP induced the marked depolarization of the mitochondrial membrane, as indicated by a significant reduction in the JC-1 aggregate/monomer ratio (15 ± 5% and 21 ± 1%, respectively, vs. 100 ± 10% in control; **** *p* < 0.0001). This depolarization was partially reversed in cells exposed to PEMFs, with the JC-1 ratios increasing to 56 ± 11% and 42 ± 2%, respectively (vs. 100 ± 10% in control, ^##^ *p* < 0.01 and ^####^ *p* < 0.0001, respectively; ^§^ *p* < 0.05, ^†^ *p* < 0.05), confirming the protective effect of PEMF exposure observed in microscopy (Figure 4C).

### 2.6. Molecular Mechanisms of PEMFs’ Effects on Phospho/Total ERK1/2 Ratio in H_2_O_2_- and CP-Injured PC12 Cells

**Figure 5:**
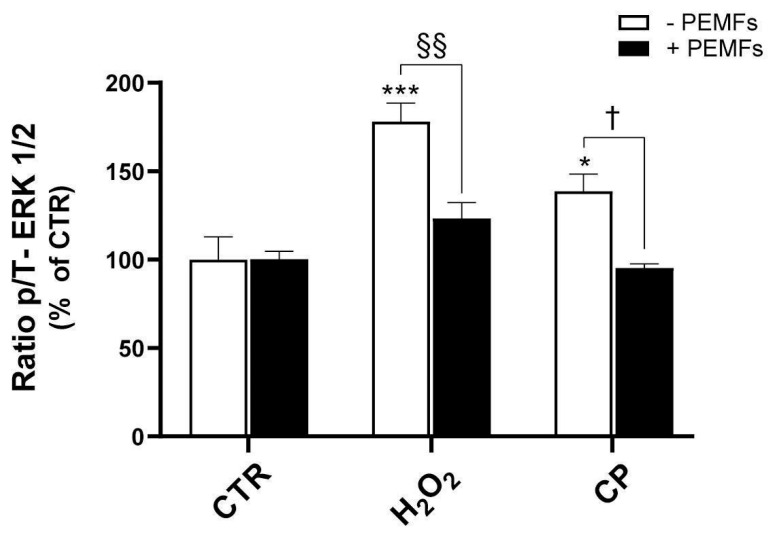
PEMFs’ effects on ERK1/2 in PC12 cells. Quantification of total (T) and phosphorylated (p) forms of ERK1/2 after 1 mM H_2_O_2_ or 20 μM CP treatment of cells for 20 min. Results are presented as mean ± SEM values of at least three independent experiments performed in duplicate (*** *p* < 0.001 and * *p* < 0.05 vs. control (CTR) without PEMFs; ^§§^ *p* < 0.01; ^†^ *p* < 0.05). Statistical analysis was performed by one-way ANOVA and Sidak’s multiple comparison test.

Since the MEK ERK1/2 inhibitor U0126 was found to have a protective effect on the cytotoxicity induced by H_2_O_2_ and CP, we further investigated the activation status of the ERK1/2 signaling pathway under these conditions. To this aim, we studied the expression of the total and phosphorylated forms of ERK1/2 in injured cells, with and without PEMF exposure. As shown in Figure 5, 1 mM H_2_O_2_ and 20 μM CP treatment for 20 min markedly upregulated the p/T-ERK1/2 ratio (178 ± 11% and 139 ± 10% versus 100 ± 13%, *** *p* < 0.001 and * *p* < 0.05, respectively), indicating the activation of the ERK1/2 signaling pathway in response to these stressors. Moreover, the exposure of cells to PEMFs significantly decreased the p-/T-ERK1/2 ratio in both H_2_O_2_- and CP-treated cells (123 ± 9% and 95 ± 2%, ^§§^ *p* < 0.01 and ^†^ *p* < 0.05, respectively), leading to values closer to those observed in control conditions. These findings suggest that PEMFs are able to counteract the aberrant activation of the ERK1/2 pathway induced by H_2_O_2_- and CP-related stress, possibly contributing to their overall protective effects.

### 2.7. PEMFs’ Effects on Phospho/Total CREB Ratio, cAMP, and BDNF Levels in PC12 Cells

**Figure 6:**
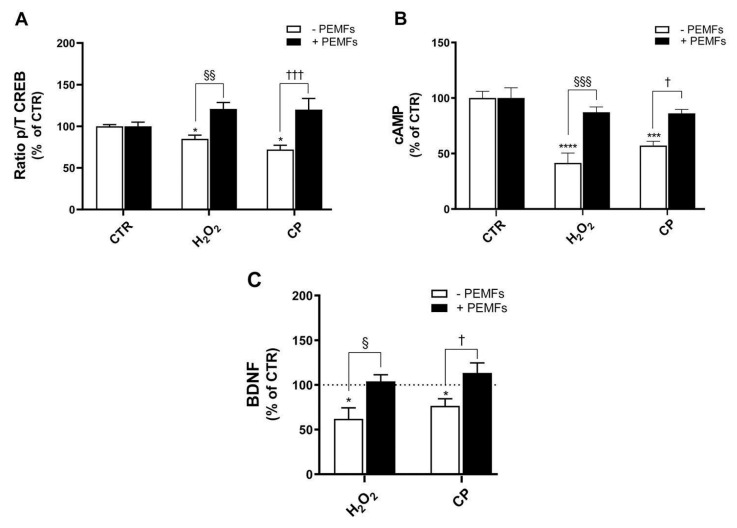
PEMFs’ effects on CREB, cAMP, and BDNF in PC12 cells. (**A**) Total (T) and phosphorylated (p) forms of CREB after 1 mM H_2_O_2_ or 20 μM CP treatment of cells for 20 min. Results are presented as mean ± SEM values of at least four independent experiments performed in duplicate (* *p* < 0.05 vs. control (CTR) without PEMFs; ^§§^ *p* < 0.01; ^†††^ *p* < 0.001). (**B**) cAMP production after 1 mM H_2_O_2_ and 20 μM CP cell treatment. Results are presented as mean ± SEM values of at least three independent experiments performed in duplicate (**** *p* < 0.0001 and *** *p* < 0.001 vs. CTR without PEMFs, respectively; ^§§§^ *p* < 0.001; ^†^ *p* < 0.05). (**C**) Extracellular BDNF levels after 200 μM H_2_O_2_ or 20 μM CP treatment for 24 h. Results are presented as mean ± SEM values of at least three independent experiments performed in duplicate (* *p* < 0.05 vs. CTR without PEMFs; ^§^ *p* < 0.05; ^†^ *p* < 0.05). Statistical analysis was performed by one-way ANOVA and Sidak’s multiple comparison test. cAMP, cyclic adenosine monophosphate; CREB, cAMP response element-binding protein; BDNF, brain-derived neurotrophic factor.

To further investigate the role of CREB in our injured cell model, and since the 666-15 CREB inhibitor aggravated H_2_O_2_- and CP-induced cell death, we studied the expression of total and phosphorylated CREB. A 20 min treatment with 1 mM H_2_O_2_ or 20 μM CP significantly reduced the phospho/total CREB ratio compared to the control (85 ± 4% and 72 ± 5%, respectively, vs. 100 ± 2%; * *p* < 0.05), indicating the downregulation of CREB activation following both oxidative and amyloidogenic stress (Figure 6A). Notably, when cells were exposed to PEMFs immediately after the injury stimuli, a significant increase in the phospho/total CREB ratio was observed (121 ± 8% and 120 ± 13%, respectively; ^§§^ *p* < 0.01, ^†††^ *p* < 0.001), suggesting a protective effect mediated through CREB activation. Given the involvement of CREB in the cellular response to injury, we investigated potential upstream mechanisms, focusing on cAMP signaling. The results included in Figure 6B show that the production of cAMP was significantly decreased after 1 mM H_2_O_2_ and 20 μM CP cell treatment (42 ± 9% and 57 ± 4% versus 100 ± 6%, **** *p* < 0.0001 and *** *p* < 0.001, respectively). Cells’ exposure to PEMFs during treatment in part rescued the decreased cAMP levels (87 ± 5% and 86 ± 4%, ^§§§^ *p* < 0.001 and ^†^ *p* < 0.05, respectively), indicating a possible link between PEMF-induced CREB phosphorylation and cAMP signaling, supporting the hypothesis that PEMFs act upstream of CREB activation. We further evaluated the functional consequences of CREB modulation by analyzing the release of BDNF, a key neurotrophic factor regulated by CREB. Cells’ treatment with 200 μM H_2_O_2_ or 20 μM CP for 24 h resulted in a significant reduction in extracellular BDNF levels (62 ± 12% and 77 ± 8%, respectively, vs. 100 ± 5% in control; * *p* < 0.05), indicating impaired neurotrophic support under stress conditions (Figure 6C). Importantly, PEMF exposure significantly increased BDNF release (104 ± 7% and 113 ± 11%, respectively; ^§^ *p* < 0.05, ^††^ *p* < 0.05), further supporting the neuroprotective role of PEMFs through the modulation of CREB and its downstream target.

### 2.8. DCF Effects on H_2_O_2_- and CP-Injured PC12 Cells

**Figure 7:**
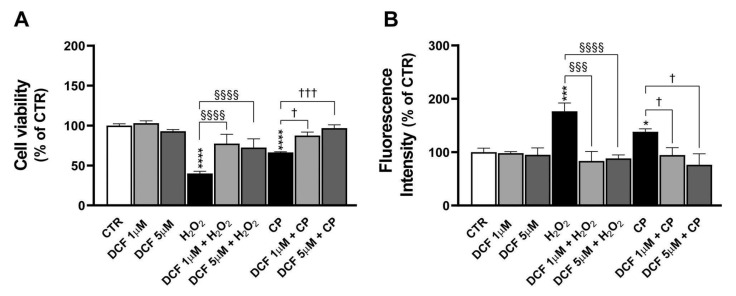
Diclofenac (DCF) effects in injured PC12 cells. Cell viability and ROS production were studied in cells pretreated for 30 min with 1 μM or 5 μM DCF and then treated with 1 mM H_2_O_2_ or 20 μM CP for 24 h, through MTS (**A**) and H_2_DCFDA (**B**) assays. (**A**) Results are presented as mean ± SEM values of at least four independent experiments performed in duplicate (**** *p* < 0.0001 vs. control, CTR; ^§§§§^ *p* < 0.0001; ^†^ *p* < 0.05 and ^†††^ *p* < 0.001). (**B**) The graph shows mean ± SEM values from three independent experiments performed in duplicate (*** *p* < 0.001 and * *p* < 0.05 vs. CTR; ^§§§^ *p* < 0.001 and ^§§§§^ *p* < 0.0001; ^†^ *p* < 0.05). Statistical analysis was performed by one-way ANOVA and Sidak’s multiple comparison test. MTS, 3-(4,5-dimethylthiazol-2-yl)-5-(3-carboxymethoxyphenyl)-2-(4-sulfophenyl)-2H-tetrazolium.

The effect of DCF, a non-steroidal anti-inflammatory drug (NSAID), on injured H_2_O_2_ and CP cells was evaluated. Cells were pretreated with DCF at concentrations of 1 μM or 5 μM for 30 min prior to 24 h exposure to 1 mM H_2_O_2_ or 20 μM CP. As can be observed in Figure 7A, both DCF concentrations conferred partial protection against H_2_O_2_-induced cytotoxicity. In particular, cell viability increased from 40 ± 3% in H_2_O_2_-treated cells to 78 ± 12% and 73 ± 11% after pretreatment with 1 μM and 5 μM DCF, respectively (^§§§§^ *p* < 0.0001 for both comparisons). A protective effect of DCF was also observed in CP-treated cells, where the viability increased from 66 ± 1% (CP alone) to 88 ± 4% with 1 μM DCF (^†^ *p* < 0.05) and to 97 ± 4% with 5 μM DCF (^†††^ *p* < 0.001), suggesting a dose-dependent cytoprotective effect of DCF. Regarding ROS production, treatment with H_2_O_2_ and CP induced a significant increase compared to the control (177 ± 16% and 138 ± 5% versus 100 ± 8%, *** *p* < 0.001 and * *p* < 0.05, respectively). In the same way, cells pretreated with DCF at 1 and 5 μM showed inhibited ROS production in response to H_2_O_2_ (84 ± 18% and 88 ± 7%, ^§§§^ *p* < 0.001 and ^§§§§^ *p* < 0.0001, respectively) and to CP (95 ± 14% and 76 ± 21%, respectively, ^†^ *p* < 0.05) (Figure 7B), indicating an antioxidant effect.

## 3. Discussion

The aim of this study was to investigate the protective effects of PEMFs on rat neuron-like PC12 cells insulted with H_2_O_2_ and Aβ peptide in order to mimic key aspects of AD pathophysiology. Due to their neuronal characteristics and oxidative stress sensitivity, PC12 cells are widely used as an in vitro model for the study of AD-related mechanisms [23]. H_2_O_2_ is a powerful oxidative stressor producing ROS in PC12 cells, thus exacerbating oxidative damage [24,25]. Similarly, Aβ peptide contributes to the formation of senile plaques and induces oxidative stress through mitochondrial dysfunction and other cellular pathways. In particular, in PC12 cells, Aβ exposure leads to elevated ROS production, which in turn promotes cellular damage and apoptosis [26,27].

The present work offers a comprehensive view of the neuroprotective action of PEMFs, acting via the orchestrated modulation of multiple signaling pathways in NGF-differentiated PC12 cells exposed to oxidative and amyloidogenic insults. The data highlight that PEMFs do not act through a single molecular entity but through multitarget signaling that stabilizes the redox balance, suppresses pro-death agents, and activates neurotrophic pathways.

In particular, this study found that PEMF exposure significantly reduced neuronal cell death induced by H_2_O_2_ and CP in PC12 cells. As for the mechanistic aspect of their protection, it was found that the damage caused by these insults was at least in part recovered by a caspase-3 inhibitor and by Trolox, a known antioxidant agent, suggesting that the protective effects of PEMFs may be mediated through the modulation of apoptotic pathways and oxidative stress.

Caspase-3 is a key enzyme in the apoptotic pathway and serves as a critical marker in neurodegenerative diseases such as AD. In our study, the use of a caspase-3 inhibitor protected injured PC12 cells from cell death, suggesting the involvement of this apoptotic protein in the observed damage. To gain deeper insights into this mechanism, we analyzed cleaved caspase-3 protein expression, which was significantly upregulated in cells treated with H_2_O_2_ and CP, confirming its role in the programmed cell death pathways activated in our model. At the nuclear level, PEMFs suppressed caspase-3 activation and reduced chromatin condensation, demonstrating the blockade of the executioner phase of apoptosis. These findings align with prior in vitro and in vivo evidence demonstrating decreased neuronal or neuroblastoma cell death, reduced infarct volumes, and enhanced recovery following extremely low-frequency magnetic field (ELF-MF) or PEMF therapy in experimental stroke models [28,29,30,31,32]. Similar results were found following chronic exposure to 50 Hz ELF-MFs in primary rat cortical neurons, showing enhanced cell viability and reduced apoptosis without inducing overt oxidative damage. Although the ROS levels showed a slight increase, this was effectively counterbalanced by intracellular glutathione, whose levels correlated inversely with apoptosis and directly with ROS, suggesting dynamic redox adaptation [33].

Trolox, a water-soluble analog of vitamin E, is well known for its potent antioxidant properties. It exerts protective effects on H_2_O_2_-treated PC12 cells through multiple mechanisms, including the direct scavenging of ROS, attenuation of oxidative stress, and prevention of cellular damage. Additionally, Trolox inhibits lipid peroxidation, thereby preserving membrane integrity, and enhances endogenous antioxidant defenses by upregulating antioxidant enzymes [34]. Consistent with previous findings in the AD mouse model, our results show that Trolox partially mitigated the cytotoxic effects induced by H_2_O_2_ or CP in PC12 cells, supporting the involvement of oxidative stress in the observed cell death [35]. Specifically, exposure to PEMFs significantly reduces oxidative stress. This is evidenced by decreased levels of reactive oxygen species (ROS) and the restoration of catalase activity, indicating that the treatment acts upstream in the oxidative cascade. This result was confirmed in SH-SY5Y cells damaged with H_2_O_2_ or CP, where high-frequency low-intensity pulsed electric fields (H-LIPEFs) as well as PEMFs were able to reduce ROS [15,18]. Furthermore, recent studies have reinforced the neuroprotective potential of PEMFs across various models of neurodegeneration. For instance, it has been demonstrated that PEMFs modulate mitochondrial and oxidative stress-related pathways in SH-SY5Y cells within a Parkinson’s disease model, using a metabolomic approach to highlight conserved cellular responses to PEMF exposure [36]. Similarly, PEMFs exerted indirect neuroprotective effects by stimulating vascular endothelial growth factor release from human astrocytes, which in turn protected SH-SY5Y neurons from ischemic-like injury [37]. This suggests that PEMFs may act on multiple cellular components of the neurovascular unit, including glial cells, to promote neuronal resilience. Taken together with our findings in PC12 cells, these studies underscore the robustness and versatility of PEMF-induced neuroprotection across different neuronal models and pathological contexts.

While our study focused on CAT activity as a marker of antioxidant defense, other enzymes, such as superoxide dismutase (SOD) and glutathione peroxidase (GPx), also play crucial roles in cellular redox homeostasis. CAT was selected due to its direct role in detoxifying H_2_O_2_, the primary oxidative agent used in our model. This choice was supported by previous studies demonstrating CAT’s sensitivity to oxidative stress in PC12 cells [38,39]. Nonetheless, future investigations will include a broader panel of antioxidant enzymes to fully elucidate the spectrum of PEMF-induced antioxidant responses. Indeed, it has been reported that PEMFs enhance mitochondrial superoxide dismutase (MnSOD) activity and reduce ROS accumulation in drug-resistant SK-N-BE(2) neuroblastoma cells exposed to H_2_O_2_, further supporting their role in oxidative stress mitigation [40].

In PC12 cells, exposure to H_2_O_2_ results in the significant loss of MMP, indicating impaired mitochondrial function [38]. This disruption of MMP can trigger the release of cytochrome c from mitochondria to the cytosol, activating caspases and promoting apoptosis [41]. On the other hand, Aβ in mitochondria specifically can directly induce apoptotic cell death by disrupting mitochondrial function [42]. We employed the JC-1 assay to evaluate MMP as an indicator of mitochondrial integrity [43]. Our results demonstrated that treatment with H_2_O_2_ and CP led to a significant reduction in MMP. However, exposure to PEMFs partially mitigated this effect. In the groups subjected to PEMFs during injury treatments, an increased presence of JC-1 aggregates (dimers), which colocalized with monomers, was observed, suggesting a protective effect on mitochondrial function. The preservation of MMP further confirms the centrality of mitochondrial protection in PEMF-mediated effects. These findings align with prior studies showing that PEMFs or H-LIPEFs mitigate mitochondrial dysfunction and apoptosis in SH-SY5Y cells exposed to H_2_O_2_ or Aβ [15,44].

Existing research indicates that ERK1/2 is dysregulated in AD patients [9]. In particular, the altered APP expression and thus Aβ production cause the activation and phosphorylation of ERK1/2 proteins, with potential implications for AD pathology, including tau phosphorylation and neuroinflammation [9]. Our findings confirm that the exposure of PC12 cells to the toxic agents H_2_O_2_ and CP results in a marked increase in ERK1/2 phosphorylation, suggesting the activation of stress-related signaling pathways. Interestingly, treatment with PEMFs effectively counteracted this effect, reducing ERK1/2 activation, as shown by the reduced phosphorylation of ERK1/2. The pharmacological inhibition of MEK with U0126 produced partial protection, reinforcing the contribution of ERK in the cytotoxic response. These data confirm a trend already observed in SH-SY5Y cells insulted with H_2_O_2_ or Aβ, where stimuli were applied for a short period of time, such as 20 min [15]. However, it is important to note that the role of ERK signaling in neuronal survival is highly context-dependent and not always pro-apoptotic. Indeed, H-LIPEFs applied to SH-SY5Y cells exposed to H_2_O_2_ or Aβ for 24 h resulted in the activation of the ERK pathway, which was associated with neuroprotection [18,44]. These findings highlight the complex and time-dependent nature of ERK signaling in neuronal cells, suggesting that its modulation by PEMFs may have different outcomes depending on the cellular context and timing of activation.

In addition, evidence from the literature reports CREB to be involved in neurodegenerative diseases. In fact, its downregulation is related to the pathology of AD, since reduced CREB phosphorylation results in lower transcriptional activity, which in turn affects synaptic plasticity and neuronal loss. Thus, increasing CREB expression has been considered as a potential therapeutic strategy for AD [45,46]. In PC12 cells, the inhibition of CREB has been described to reduce the protective effects of neurotrophic factors, making cells more susceptible to oxidative stress and Aβ-induced damage [47]. PEMFs reactivated the cAMP/CREB/BDNF axis, a key neurotrophic signaling pathway typically impaired under oxidative or amyloidogenic stress. Our experiments showed that PEMFs restored the intracellular cAMP levels, increased CREB phosphorylation, and enhanced BDNF secretion. The CREB inhibitor 666-15 increases cell death induced by H_2_O_2_ and CP, confirming the role of CREB as a downstream effector. Accordingly, in SH-SY5Y cells, the application of H-LIPEFs activated the ERK/CREB pathway, producing protective pro-survival effects [18,44]. Notably, PEMF exposure activated BDNF/TrkB/Akt signaling in ischemic mice, contributing to synaptic recovery and functional improvement [48]. Furthermore, the ability of PEMFs to simultaneously enhance cAMP, p-CREB, and BDNF expression while downregulating ERK1/2 activity highlights their potential to provide neuroprotective effects. This dynamic interplay underscores the importance of targeting multiple signaling pathways to restore neuronal homeostasis and support cognitive function. Indeed, several signaling mechanisms are potentially modulated by PEMFs, and future studies will be aimed at investigating additional pathways, including PI3K/Akt and NF-κB, to further elucidate the multifaceted molecular effects of PEMFs.

Finally, this study also compared the effects of PEMFs with those of DCF, a widely used non-steroidal anti-inflammatory drug (NSAIDs). DCF exerts its anti-inflammatory action by inhibiting cyclooxygenase enzymes, thereby reducing the synthesis of pro-inflammatory prostaglandins. This mechanism directly targets the inflammatory cascade, which is a key contributor to neurodegeneration [49]. In contrast, PEMFs act through the biophysical modulation of intracellular signaling pathways, including the suppression of ERK1/2 activation and the enhancement of the cAMP/CREB/BDNF axis, as shown in our study. These distinct modes of action suggest that PEMFs and NSAIDs could be used in combination to achieve broader neuroprotection. Future studies should investigate the potential synergistic or additive effects of such combined approaches, which may offer enhanced efficacy while minimizing the dosages and side effects of pharmacological agents. Compared to current pharmacological treatments for AD, such as Lecanemab, a monoclonal antibody targeting amyloid-beta, PEMFs offer a non-invasive and potentially safer alternative. While Lecanemab has demonstrated modest efficacy in slowing cognitive decline in early AD, its use is limited by high costs, intravenous administration, and the risk of amyloid-related imaging abnormalities, which require regular magnetic resonance imaging monitoring [50,51]. In contrast, PEMFs are well tolerated, do not require systemic delivery, and have shown neuroprotective effects through the modulation of multiple signaling pathways. Although further preclinical and clinical validation is needed, PEMFs may represent a valuable adjunct or alternative to current therapies, especially in long-term management scenarios.

## 4. Materials and Methods

### 4.1. Materials

Penicillin–streptomycin (10,000 units penicillin–10 mg streptomycin/mL), 200 mM L-glutamine, RPMI-1640 medium, Dulbecco’s modified eagle’s medium high-glucose (DMEM), Hanks’ balanced salt solution (HBSS), 4-(2-hydroxyethyl)piperazine-1-ethanesulfonic acid (HEPES (CAS N. 7365-45-9), H_2_O_2_ solution, phosphate-buffered saline (PBS), anhydrous DMSO, 0.01% poly-L-lysine, diclofenac (DCF) (CAS N. 15307-79-6), bovine serum albumin (BSA), forskolin (CAS N. 66575-29-9), RO (CAS N. 29925-17-5), Trolox (CAS N. 53188-07-1), and rat recombinant NGF were purchased from Merck (Milan, Italy). Donor horse serum and fetal bovine serum were purchased from EuroClone (Milan, Italy). Caspase-3 inhibitor was purchased from Merck (Milan, Italy). The ROS detection assay kit (dichlorofluorescin diacetate/2′,7′-dichlorodihydrofluorescein diacetate; DCFDA/H_2_DCFDA) was from Canvax Biotech (Voden, MB, Italy). The CellTiter 96^®®^ AQueous One Solution MTS Cell Proliferation Assay was from Promega (Milan, Italy). The mitochondrial membrane potential (MMP) assay kit (ab113850 JC-1: 5,5′,6,6′-tetrachloro-1,1′,3,3′-tetraethylbenzimidazolylcarbocyanine iodide) was from Abcam (Cambridge, UK). The catalase colorimetric activity kit (EIACATC) and multispecies ERK1/ERK2 (ERK1/2) (total/phospho) InstantOne™ ELISA kit (catalog number: 85-86013) were from ThermoFisher Scientific (Milan, Italy). The rat BDNF enzyme-linked immunosorbent (ELISA) assay kit (catalog number: ELK5459) was from ELK Biotechnology (Denver, CO, USA). Click-peptide Aβ_1-42_ (CP) was from GenScript Biotech (Twin Helix, Milan, Italy). Rat pheochromocytoma PC12 cells were purchased from the American Type Culture Collection (Manassas, VA, USA). Rabbit anti-caspase-3 (Cleaved Asp175) polyclonal antibody and goat anti-rabbit IgG (H+L) cross-adsorbed secondary antibody Alexa Fluor™ 488 were purchased from ThermoFisher Scientific (Milan, Italy). Paraformaldehyde (4%) solution and Triton X-100 were from Merck (Milan, Italy). The Alpha Screen cyclic adenosine monophosphate (cAMP) detection kit (catalog number: 6760635D) and AlphaLISA™ SureFire^®®^ Ultra™ total (catalog number: ALSU-TCREB-A-HV) and phospho-CREB (catalog number: ALSU-PCREB-A-HV) were from Revvity (Milan, Italy).

### 4.2. Electromagnetic Field Exposure System

The PEMF exposure system has already been described in detail [52]. PC12 cells were exposed to PEMFs generated by a pair of rectangular coils (14 × 23 cm), composed of 1400 turns of copper wire placed opposite to each other. The cells were placed between coils so that the plane of the coils was perpendicular to the culture plate. The coils were powered by the PEMF generator system (IGEA, Carpi, Italy). The selection of PEMF parameters (pulse duration 1.3 ms, frequency 75 Hz, magnetic field intensity 1.5 ± 0.2 mT) was based on previous studies using the same exposure system in neuronal and glial models [14,15,52]. In particular, both 1.5 mT and 3.0 mT PEMFs, applied with identical pulse characteristics, induced consistent biological effects [52]. The 1.5 mT intensity was selected in the present study as it represents the lowest effective dose capable of eliciting neuroprotective responses, ensuring both biological efficacy and safety. The peak intensity of the magnetic field and peak intensity of the induced electric voltage were detected in air between two coils from one side to the other, at the level of the culture plate. The peak values measured between two coils in air had a maximum variation of 1% in the whole area in which the culture plates were placed. The peak intensity of the magnetic field was detected using the Hall probe (HTD61-0608-05-T, 8 F.W. Bell, Sypris Solutions, Louisville, KY, USA) of a gaussmeter (DG500, Laboratorio Elettrofisico, Milan, Italy), with reading sensitivity of 0.2%. The corresponding peak amplitude of the induced electric voltage was 2.0 ± 0.5 mV. It was detected using a standard coil probe (50 turns, 0.5 cm internal diameter of the coil probe, 0.2 mm copper diameter), and the temporal pattern of the signal was displayed using a digital oscilloscope (Le Croy, Chestnut Ridge, NY, USA). The shape of the induced electric voltage and its impulse length were kept constant.

### 4.3. Cell Cultures

PC12 cells were cultured in RPMI-1640 medium supplemented with 2 mM L-glutamine, 1% (*v/v*) penicillin–streptomycin, 10% (*v/v*) horse serum (HS), and 5% (*v/v*) fetal bovine serum (FBS) at 37 °C in a 5% CO_2_-humidified incubator. For neuron-like induced differentiation, PC12 cells were cultured in DMEM high-glucose medium supplemented with 2 mM L-glutamine, 1% (*v/v*) penicillin–streptomycin, 10% (*v/v*) HS, and 5% (*v/v*) FBS containing 100 ng/mL of NGF and maintained in these conditions (differentiation medium) for 3 days. All experiments included in this study were performed with NGF-differentiated PC12 cells. Before the experiments, to perform appropriate treatments, the differentiation medium was replaced with serum-free fresh differentiation medium [53]. PC12 cells, although responsive to NGF and capable of acquiring neuron-like properties, do not fully replicate the complexity of primary neurons or the in vivo environment. They lack the full repertoire of synaptic connections, glial interactions, and genetic background relevant to AD pathology. In addition, their response to stimuli may differ from that of primary neurons or transgenic animal models, which should be employed in future studies.

### 4.4. Cell Treatments

In order to mimic cell insult, two types of compounds were used: (i) H_2_O_2_ (200 or 1000 μM), an oxygen radical causing neuronal toxicity, and (ii) CP (20 μM) for the induction of toxicity and inflammation. Cells were treated with H_2_O_2_ or CP, in the presence or the absence of PEMFs. The exposure times were carefully chosen based on preliminary dose–response studies that identified optimal 24 h damage conditions using 1 mM H_2_O_2_ and 20 µM CP. The durations were also adapted to the expected kinetics of the biological processes investigated: 90 min for acute effects like mitochondrial membrane potential depolarization (JC-1 test) and chromatin alterations (Hoechst 33342 staining), 20 min for rapid molecular responses such as ERK1/2 and CREB phosphorylation, 1 h for cyclic AMP (cAMP) quantification, and 4 h for catalase enzyme activity.

### 4.5. Preparation of CP Stocks

CP possesses an ester bond at the Gly (25)-Ser (26) sequence. It is a water-soluble and non-aggregative precursor molecule that is promptly converted to Aβ_1-42_ (t_1/2_ 10 s) at pH 7.4 via an O-to-N intramolecular acyl migration reaction. It was prepared as already described [15]. To ensure accurate interpretation, the effects observed in this study should be considered as attributable to CP, rather than to physiological Aβ_1-42_ or its aggregated forms.

### 4.6. MTS Assay

The 3-(4,5-dimethylthiazol-2-yl)-5-(3-carboxymethoxyphenyl)-2-(4-sulfophenyl)-2h-tetrazolium (MTS) assay was performed to determine cell viability, following the manufacturer’s instructions. In brief, 3.1 × 10^4^ cells/cm^2^ were seeded in 96-well plates (Nunc™ MicroWell™ 96-well microplate, Thermo Fisher Scientific) and cultured for 3 days in differentiation medium. On the day of the assay, the medium was removed, and cell treatments were performed in serum-free differentiation medium. For the PEMF-exposed group of cells, the culture plate was positioned inside the PEMF exposure system for 4 h before treatments and then for 24 h during the compound’s treatment time. At the end of the incubation period, 20 μL of MTS solution was added to each well, they were incubated for 1 h, and the optical density was read using the EnSight multimode plate reader (Perkin Elmer, Milan, Italy) at 490 nm.

### 4.7. ROS Detection Assay (DCFDA/H_2_DCFDA)

ROS production in PC12 cells was tested by the H_2_DCFDA assay. A total of 3.1 × 10^4^ cells/cm^2^ were seeded in black 96-well plates (Nunc™ MicroWell™ 96-well, Thermo Fisher Scientific) and cultured for 3 days in differentiation conditions. After removing the medium, 100 μL of 20 μM H_2_DCFDA solution was added to each well. The plate was kept in the incubator for 1 h and subsequently washed once with 1X assay buffer. Thereafter, treatments were performed in serum-free differentiation medium, and the fluorescence was read after 24 h with the EnSight multimode plate reader (Perkin Elmer, Milan, Italy) (excitation 485 nm, emission 530 nm). For the PEMF-exposed group of cells, the culture plate was positioned inside the PEMF exposure system during the compound’s treatment time.

### 4.8. ERK1/2 Assay

The total and phospho ERK1/2 protein levels were determined by the InstantOne^TM^ ELISA kit specified in the Section 4.1, following the manufacturer’s instructions. Cells were seeded in 24-well plates at a density of 1 × 10^5^ cells/cm^2^ and cultured for 3 days in differentiation medium. On the day of the assay, the medium was changed for serum-free differentiation medium and cells were treated with 1 mM H_2_O_2_ or 20 μM CP for 20 min. Cell extracts were prepared using the specific lysis buffer provided in the kit. In brief, samples were incubated with the total or phosphor ERK1/2 capture/detection antibody cocktail (1 h at RT). After washing, the wells were incubated with the detection solution for 20 min at RT, and, finally, the stop solution was added. Wells were read at a 450 nm wavelength with the EnSight multimode plate reader (Perkin Elmer, Milan, Italy).

### 4.9. JC-1 Test

The JC-1 MMP assay kit was used for the measurement of the mitochondrial membrane potential (MMP, Δψm). This kit contains tetraethyl benzimidazolyl carbocyanine iodide (JC-1), a cationic dye that accumulates in mitochondria. At high concentrations (due to high MMP), JC-1 aggregates, yielding a red-to-orange-colored emission (590 ± 17.5 nm). At low concentrations (due to low MMP), the dye is predominantly a monomer that yields green fluorescence with emission at 530 ± 15 nm. Therefore, a decrease in the aggregate red fluorescent signal is indicative of depolarization, whereas an increase is indicative of hyperpolarization.

Cells were seeded in black 96-well plates at 3.1 × 10^4^ cells/cm^2^ and cultured for 3 days in differentiation conditions. The assay was performed following the manufacturer’s instructions. Briefly, on the day of the assay, the medium was removed and 100 μL of 20 μM JC-1 was added to each well. Cells were kept in the incubator for 30 min and subsequently washed twice with 1X dilution buffer. Then, treatments were performed in serum-free differentiation medium and lasted for 90 min. For the PEMF-exposed group of cells, the culture plate was positioned inside the PEMF exposure system during compounds’ treatment. At the end of the incubation time, plates’ fluorescence was read using the EnSight multimode plate reader (Perkin Elmer, Milan, Italy) (red aggregates: excitation 535 nm, emission 590 nm; green monomers: excitation 475 nm, emission 530 nm).

### 4.10. Microscopy Imaging of JC-1 MMP

The MMP was studied using a fluorescence microscope. For this purpose, cells were seeded in 8-well chamber slides (Millicell EZ SLIDE 8-well glass slide, Merck, Italy) at 3.6 × 10^5^ cells/cm^2^ and cultured for 3 days in NGF differentiation conditions. The assay was performed following the protocol described above. On the day of the assay, after treatment with 1 mM H_2_O_2_ or 20 μM CP for 90 min, cells were analyzed under a fluorescence microscope with TRITC and FITC filters. Red and green fluorescence images were acquired using the Nikon Eclipse 5i fluorescence microscope equipped with a cooled charge-coupled device (CCD) camera (Nikon DS-Qi1). The images were analyzed using the software NIS Elements v 5.11.

### 4.11. Immunocytochemistry

Cells were seeded onto poly-L-lysine-coated chamber slides at a density of 3.6 × 10^5^ cells/cm^2^ and cultured for 3 days in NGF medium. On the day of the assay, cells were treated in serum-free medium (1 mM H_2_O_2_ or 20 μM CP for 90 min) and then fixed with 4% paraformaldehyde for 20 min at RT. After blocking non-specific binding, as well as permeabilization by incubating cells with a 0.3% Triton X-100/2% bovine serum albumin in PBS solution for 1 h at RT, the cells were incubated with caspase-3 (cleaved Asp175) primary antibody (rabbit, 1:250) overnight at 4 °C. Cells were then washed and incubated for 1 h at RT with Alexa Fluor™ 488 goat anti-rabbit secondary antibody (1:500). For nuclear labeling, PC12 cells were stained with Hoechst 33342 solution (1 μg/mL) for 20 min at RT and then washed with PBS. Finally, the slides were mounted in an anti-fade 1,4-phenylenediamine solution in PBS–glycerol (2.5%, 1:3). In order to check for non-specific labeling of the secondary antibody, staining was tested in the absence of the primary antibody and no fluorescent signal was observed. Fluorescence images were acquired using the Nikon Eclipse 5i fluorescence microscope. The analysis of both cleaved caspase-3 and Hoechst 33342 fluorescent signals was performed on at least five different fields of each sample well using the software NIS Elements v 5.11. The total number of cells in each field analyzed was counted through Hoechst 33342 staining, as well as the number of cells with chromatin-altered nuclei.

### 4.12. Catalase (CAT) Assay

The CAT activity of PC12 cells was quantitatively measured using a catalase colorimetric kit. Cells were seeded onto a 6-well plate at a density of 5.3 × 10^5^ cells/cm^2^ and cultured for 3 days in differentiation conditions. On the day of the assay, cells were treated with 200 μM H_2_O_2_ or 20 μM CP in serum-free differentiation medium and incubated for 4 h at 37 °C in the incubator. After removing the medium, cells were scraped and collected by centrifugation at 9000× *g* for 2 min at 4 °C. The pellet was homogenized in 1X assay buffer and centrifuged at 10,000× *g* for 15 min at 4 °C to obtain the supernatant, which was conserved at −80 °C till the day of the assay. The instructions described by the kit manufacturers were followed to perform the assay.

### 4.13. BDNF Assay

The extracellular levels of BDNF were measured in the supernatants of PC12 cells using a BDNF ELISA assay kit. Cells were seeded in 24-well plates at a density of 1.6 × 10^5^ cells/cm^2^ and cultured for 3 days in differentiation medium. Then, the medium was changed for serum-free differentiation medium, and cells were treated with 200 μM H_2_O_2_ or 20 μM CP for 24 h. On the day of the assay 100 μL of each sample or standard was loaded into the wells of the plate and incubated at 37 °C for 1 h 20 min. After washing (3 times with 1X wash buffer), 100 μL of biotinylated BDNF antibody working solution (1:100 in biotinylated antibody diluent) was added to each well, and the plate was incubated at 37 °C for 50 min. After washing (3 times with 1X wash buffer), 100 μL of streptavidin–HRP working solution (1:100 in HRP diluent) was added to each well, and the plate was incubated at 37 °C for another 50 min. After washing (5 times with 1X wash buffer), 90 μL of TMB substrate solution was added to each well and they were incubated at 37 °C for 20 min in the dark. Finally, the reaction was stopped by adding 50 μL of stop solution to each well, and the plate was shaken for 1 min to mix. Absorbance was immediately measured at 450 nm using the EnSight multimode plate reader (Perkin Elmer, Milan, Italy).

### 4.14. Total/Phospho CREB Assay

The AlphaLISA™ SureFire^®^ Ultra™ assay was used to measure the phosphorylated and total CREB protein in cellular extracts. Cells were seeded in 24-well plates at a density of 1 × 10^5^ cells/cm^2^ and cultured for 3 days in differentiation medium. On the day of the assay, the medium was changed for serum-free differentiation medium, and cells were treated with 1 mM H_2_O_2_ or 20 μM CP for 20 min. After removing the medium, cell extracts were prepared using the lysis buffer provided by the kit, and the assay was performed in a half-area OptiPlate^TM^ 96 (Perkin Elmer, Milan, Italy), following the manufacturer’s instructions and avoiding direct light. In brief, 30 μL of each sample was incubated with an acceptor mix composed of reaction buffer 1, reaction buffer 2, activation buffer, and acceptor beads. After 1 h of incubation at RT, the donor mix composed of beads and diluent was added to the samples and they were incubated for 1 h at RT. The plate was then read using the EnSight multimode plate reader (Perkin Elmer, Milan, Italy). The amount of light emission measured was directly proportional to the amount of target protein present in the sample.

### 4.15. cAMP Quantification

The second messenger cAMP was quantified using the ALPHAscreen cAMP assay kit, following the manufacturer’s instructions. Cells were cultured for 3 days in differentiation medium. On the day of the assay, cells were resuspended in a stimulation buffer consisting of HBSS, 5 mM HEPES, 0.5 mM MgCl_2_, 100 μM RO, and 0.05% BSA, at pH 7.4 and counted, and a cell suspension of 10^7^ cells/mL was prepared, which was mixed with 10X acceptor beads (1:1). Reactions were performed in 96-well half-area microplates. Each sample consisted of 2.5 × 10^4^ cells in 5X acceptor beads. When appropriate, cells were treated with 1 mM H_2_O_2_ or 20 μM CP, which lasted for 1 h at 37 °C. Then, 1 μM forskolin was added, and samples were incubated for 30 min at 37 °C. Finally, the detection mix solution consisting of 41.7 nM biotin–cAMP tracer and 33.3 μg/mL streptavidin donor beads diluted in 1X immunoassay buffer was added to all sample wells and they were incubated for 1 h at 37 °C. The standard curve was prepared and processed in parallel to the samples. The plate was read using the EnSight multimode plate reader.

### 4.16. Data Analysis and Statistics

All data in the figures and text are presented as the mean ± standard error of mean (SEM) of at least three independent experiments performed in duplicate. Additionally, the same graphs including standard deviations are provided in the Supplementary File (Figures S3–S9). Data sets were examined by one-way analysis of variance (ANOVA) and Sidak’s multiple comparison test. A *p* value < 0.05 was considered a standard for statistically significant differences among the groups. Data analysis and statistical analysis were performed using the GraphPad Prism 8.0.1 software.

## 5. Conclusions

Overall, our findings suggest that PEMFs positively modulate the cAMP/CREB/BDNF signaling pathway in PC12 cells subjected to oxidative and chemical injury, thereby enhancing cell survival and promoting neuroprotection. These findings support the potential of PEMFs as a non-invasive strategy to counteract key pathological features of AD. However, PC12 cells, while useful for mechanistic studies, do not fully replicate the complexity of primary neurons or the in vivo brain environment. Future studies should validate these results in primary neuronal cultures and animal models to assess the translational relevance and long-term safety of PEMF-based interventions. Overall, our findings provide a solid foundation for the further exploration of PEMFs as a complementary or alternative approach to current pharmacological therapies for neurodegenerative diseases.

## Data Availability

All data generated or analyzed during this study are included in this article.

## Supplementary Materials

The following supporting information can be downloaded at: https://www.mdpi.com/article/10.3390/ijms26136495/s1.

## Abbreviations

The following abbreviations are used in this manuscript:

AD	Alzheimer’s Disease
Aβ	Amyloid-β Peptide
BDNF	Brain-Derived Neurotrophic Factor
cAMP	Cyclic Adenosine Monophosphate
CAT	Catalase
CP	O-Acyl Isopeptide
CREB	cAMP Response Element-Binding protein
DCF	Diclofenac
ERK	Extracellular Signal-Regulated Kinase
H_2_O_2_	Hydrogen Peroxide
MMP	Mitochondrial Membrane Potential
NGF	Nerve Growth Factor
PEMFs	Pulsed Electromagnetic Fields
ROS	Reactive Oxygen Species
