# Supplementary material for: PEMFs Restore Mitochondrial and CREB/BDNF Signaling in Oxidatively Stressed PC12 Cells Targeting Neurodegeneration
Source: Int J Mol Sci. 2025 Jul 5;26(13):6495. doi: 10.3390/ijms26136495 (PMC12250253; doi:10.3390/ijms26136495)
Supplement: Supplementary file 1 [file ijms-26-06495-s001.zip › Supplementary Figures S3-S9.pdf]

GRAPHS WITH STANDARD DEVIATION

SUPPLEMENTARY FIGURE S3

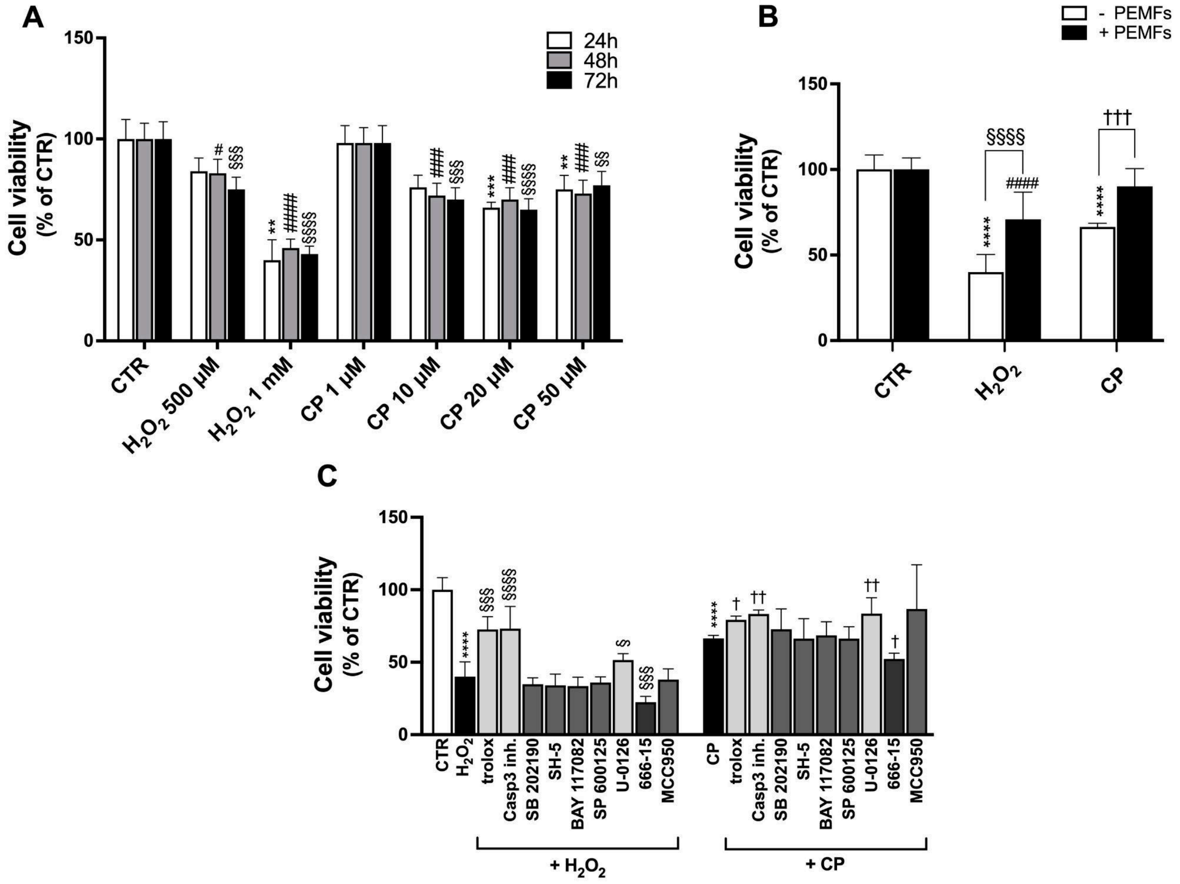

Figure S3. H_2O_2- and CP-induced cytotoxicity in PC12 cells. **A**. Cells were treated with different concentrations of H_2O_2 and CP for 24, 48 and 72h. Results are presented as mean ± SD values of at least three independent experiments performed in duplicate (**p<0.01, ***p<0.001, vs control (CTR) for 24h; $^{\#}$p<0.05, $^{\#\#\#}$p<0.001, $^{\#\#\#\#}$p<0.0001, vs CTR for 48h; §§p<0.01, §§§p<0.001, §§§§p<0.0001, vs CTR for 72h). **B**. Cell viability was studied in 1 mM H_2O_2- and 20 μM CP-injured cells for 24h in the absence and in the presence of PEMFs. Results are presented as mean ± SD values of at least seven independent experiments performed in duplicate (****p<0.0001 vs CTR without PEMFs; $^{\#\#\#\#}$p<0.0001 vs CTR with PEMFs; §§§§p<0.0001 vs H_2O_2 without PEMFs, †††p<0.001 vs CP without PEMFs). **C**. Effects of cell pre-treatment for 30 min with 500 μM trolox, 1 μM caspase-3 inhibitor, SB 202190, SH-5, BAY 117082, SP 600125, U-0126, MCC950 and 10 μM 666-15 on the viability of PC12 cells injured with 1 mM H_2O_2 or 20 μM CP. Results are presented as mean ± SD values of at least three independent experiments performed in duplicate (****p<0.0001 vs CTR; §§§§p<0.0001, §§§p<0.001, §p<0.05 vs H_2O_2-injured cells; ††p<0.01, †p<0.05 vs CP-injured cells). Statistical analysis has been performed by one-way analysis of variance (ANOVA) and Sidak's multiple comparison test. PEMFs, pulsed electromagnetic fields.

SUPPLEMENTARY FIGURE S4

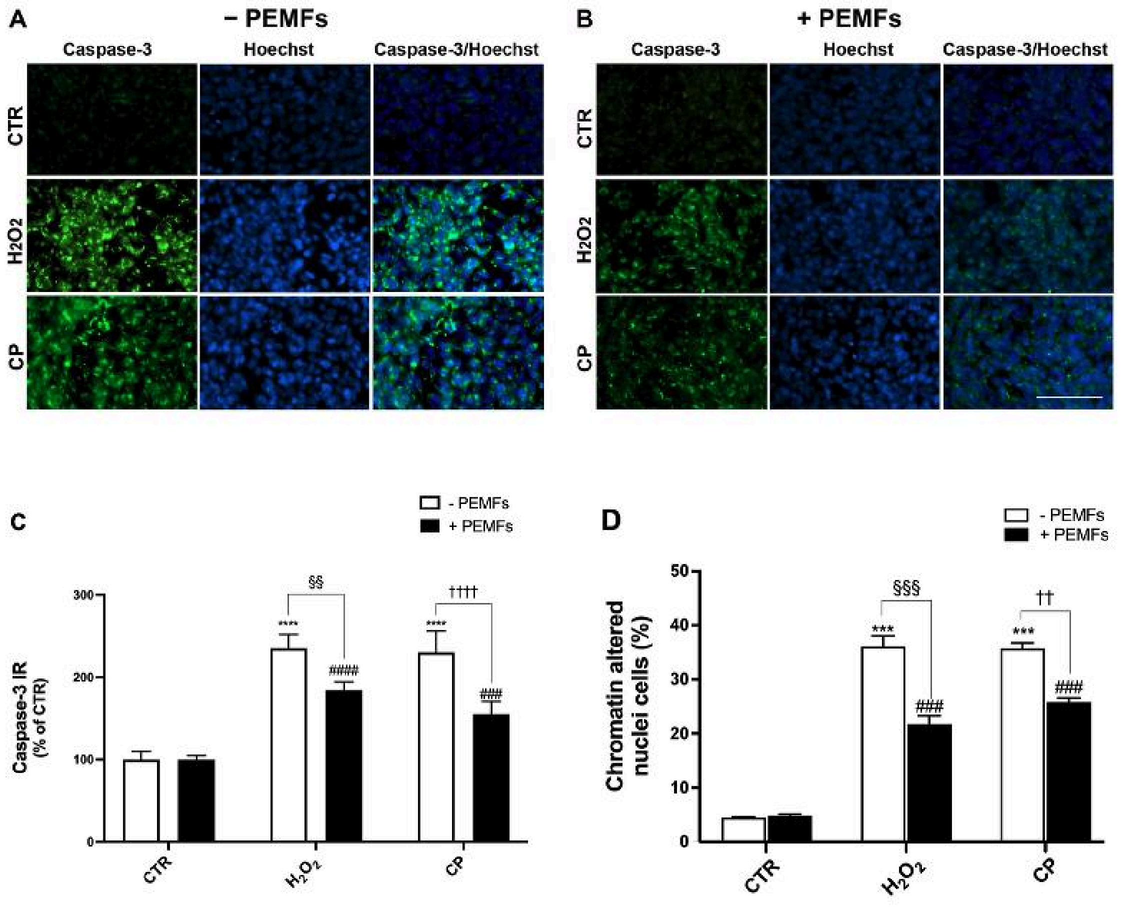

Figure S4. PEMFs' effects on cleaved caspase-3 in PC12 cells. Representative images of cleaved caspase-3 positive PC12 cells (green) in basal conditions (CTR), treated with 1 mM H_2O_2 or 20 μM CP for 90 min, without (**A**) and with (**B**) PEMFs. Hoechst 33342 nuclear staining (blue) and a merge of cleaved caspase-3/Hoechst 33342 nuclear staining have been included. Scale bar 100 μm. **C**. Analysis of cleaved caspase-3 immunoreactivity (IR) normalized to the number of cells. Results are presented as mean ± SD values of at least three independent experiments performed in duplicate (****$p<0.0001$ vs CTR without PEMFs; $^{\#\#\#\#}p<0.0001$ and $^{\#\#\#}p<0.001$ vs CTR with PEMFs, respectively; $^{\S\S}p<0.01$ vs H_2O_2-injured cells; $^{\dagger\dagger\dagger\dagger}p<0.0001$ vs CP-injured cells. **D**. Analysis of Hoechst 33342 chromatin-altered nuclei cells. Data are presented as mean ± SD values (***$p<0.001$ vs CTR without PEMFs; $^{\#\#\#}p<0.001$ vs CTR with PEMFs; $^{\S\S\S}p<0.001$ vs H_2O_2-injured cells; $^{\dagger\dagger}p<0.01$ vs CP-injured cells). Statistical analysis has been performed by one-way ANOVA and Sidak's multiple comparison test.

SUPPLEMENTARY FIGURE S5

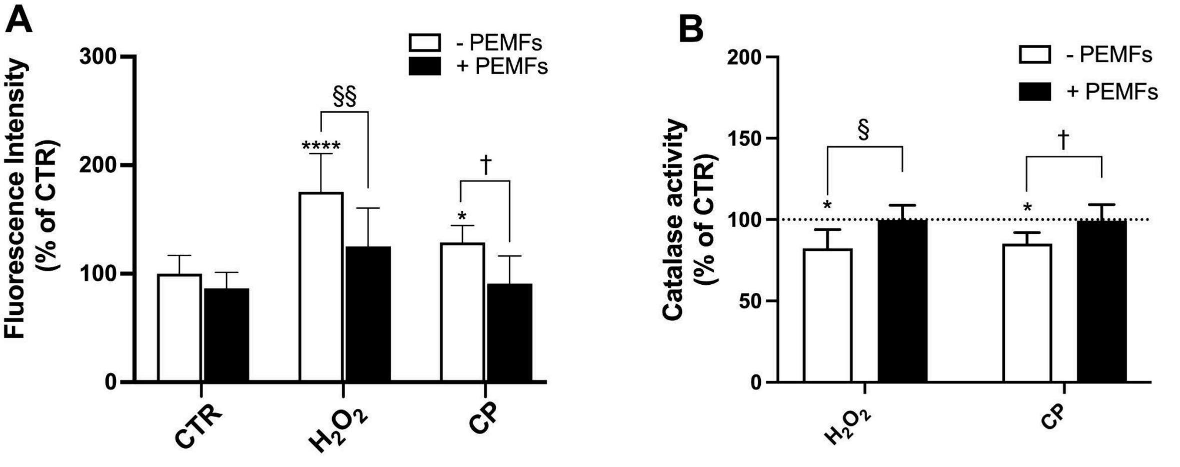

Figure S5. PEMFs' effects on oxidative stress and catalase enzyme activity in H_2O_2- and CP-injured PC12 cells. A. ROS levels in PC12 cells treated with 1 mM H_2O_2 or 20 μM CP for 24h through H_2DCFDA test. Results are presented as mean ± SD values of at least five independent experiments performed in duplicate (****p<0.0001 and *p<0.05 vs control (CTR) without PEMFs, respectively; §§p<0.01 vs H_2O_2-injured cells; †p<0.05 vs CP-injured cells). **B**. Catalase activity measured after cell treatment with 200 μM H_2O_2 or 20 μM CP for 4h. Results are presented as mean ± SD values of at least four independent experiments performed in duplicate (*p<0.05 vs CTR without PEMFs; §p<0.05 vs H_2O_2-treated cells; †p<0.05 vs CP-treated cells. Statistical analysis has been performed by one-way ANOVA and Sidak's multiple comparison test. ROS, reactive oxygen species; H_2DCFDA, 2′,7′-dichlorodihydrofluorescein diacetate.

SUPPLEMENTARY FIGURE S6

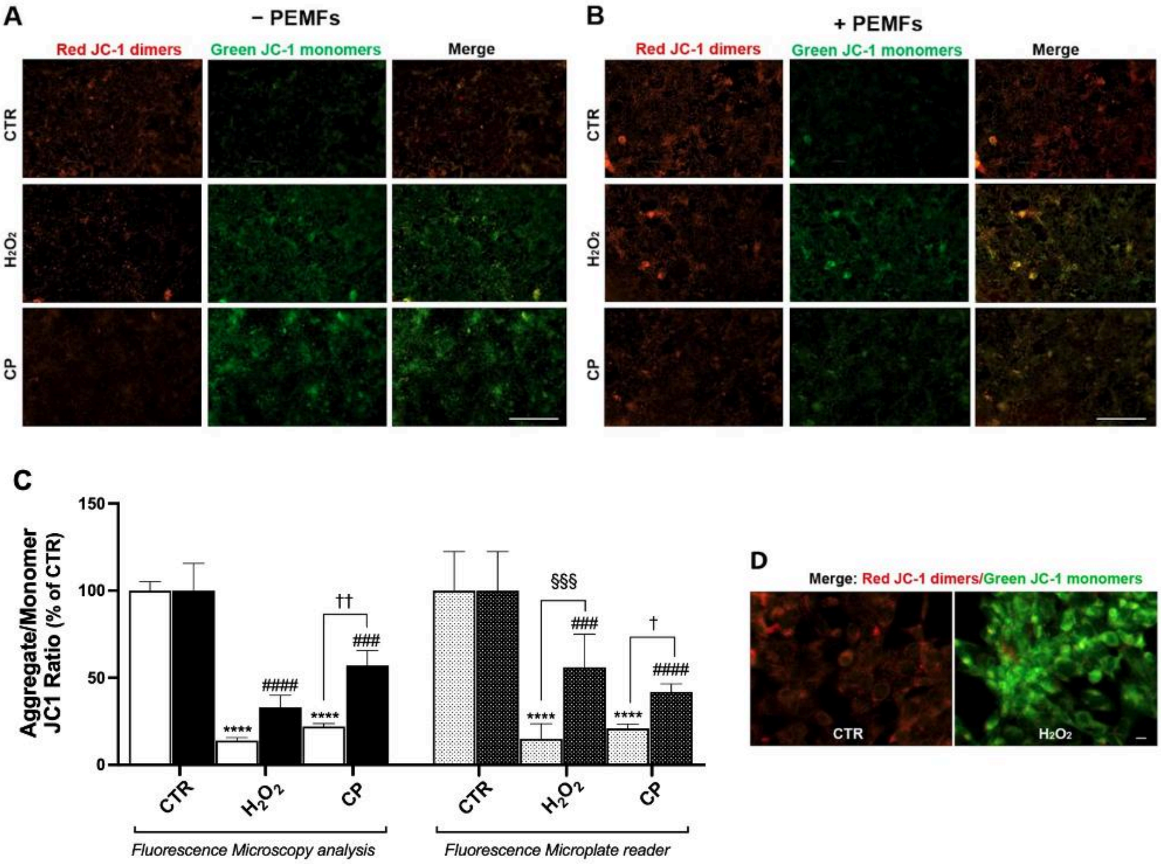

Figure S6. PEMFs' effects on MMP depolarization in PC12 cells. Representative images of JC-1 staining in 1 mM H_2O_2- or 20 µM CP-treated cells for 90 min, without PEMFs (**A**) and with PEMFs (**B**). Scale bar 100 µm. **C.** The graph includes the results of fluorescence microscope images analysis and fluorescence microplate reader analysis, expressed as the red/green *ratio* percentage of control groups (CTR). These results are presented as mean ± SD values of at least three independent experiments performed in duplicate (****p<0.0001 vs CTR without PEMFs; $^{###}$p<0.001, $^{#####}$p<0.0001 vs CTR with PEMFs; §§§p<0.05 vs H_2O_2-treated cells; †p<0.05 and ††p<0.01 vs CP-treated cells). Statistical analysis has been performed by one-way ANOVA and Sidak's multiple comparison test. **D**. Representative high-magnification images of JC-1-stained cells under CTR and after treatment with 1 mM H_2O_2. Scale bar 10 µm. MMP, mitochondrial membrane potential.

SUPPLEMENTARY FIGURE S7

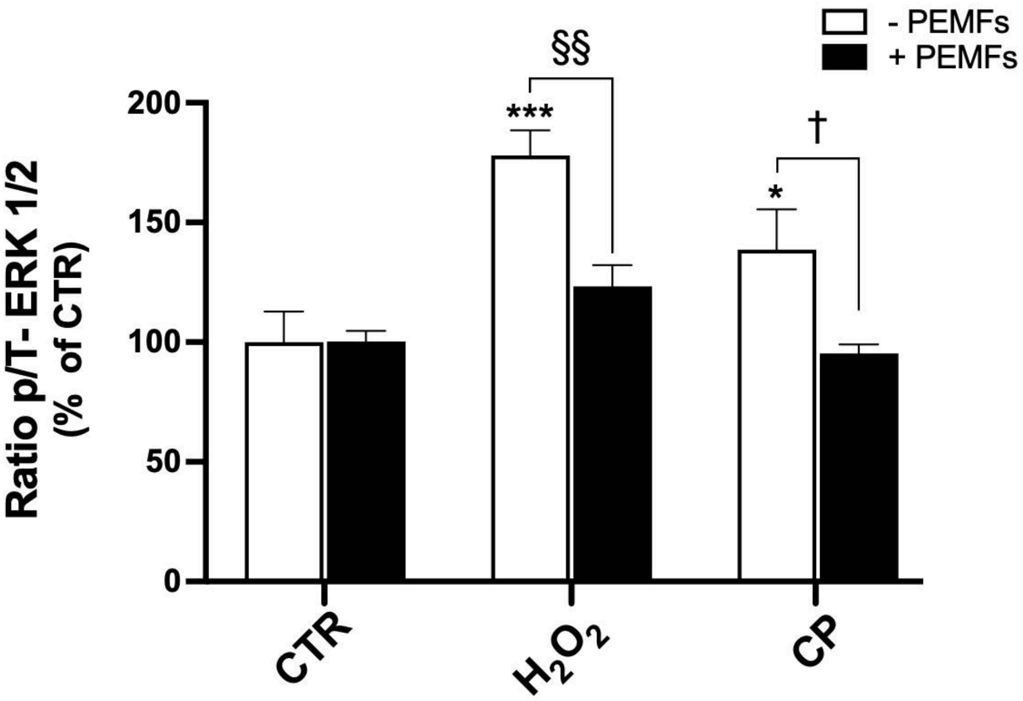

Figure S7. PEMFs' effects on ERK1/2 in PC12 cells. Quantification of total (T) and phosphorylated (p) forms of ERK1/2 after 1 mM H_2O_2 or 20 μM CP treatment of cells for 20 min. Results are presented as mean ± SD values of at least three independent experiments performed in duplicate (***p<0.001 and *p<0.05 vs control (CTR) without PEMFs; §§p<0.01 vs H_2O_2-treated cells; †p<0.05 vs CP-treated cells). Statistical analysis has been performed by one-way ANOVA and Sidak's multiple comparison test.

SUPPLEMENTARY FIGURE S8

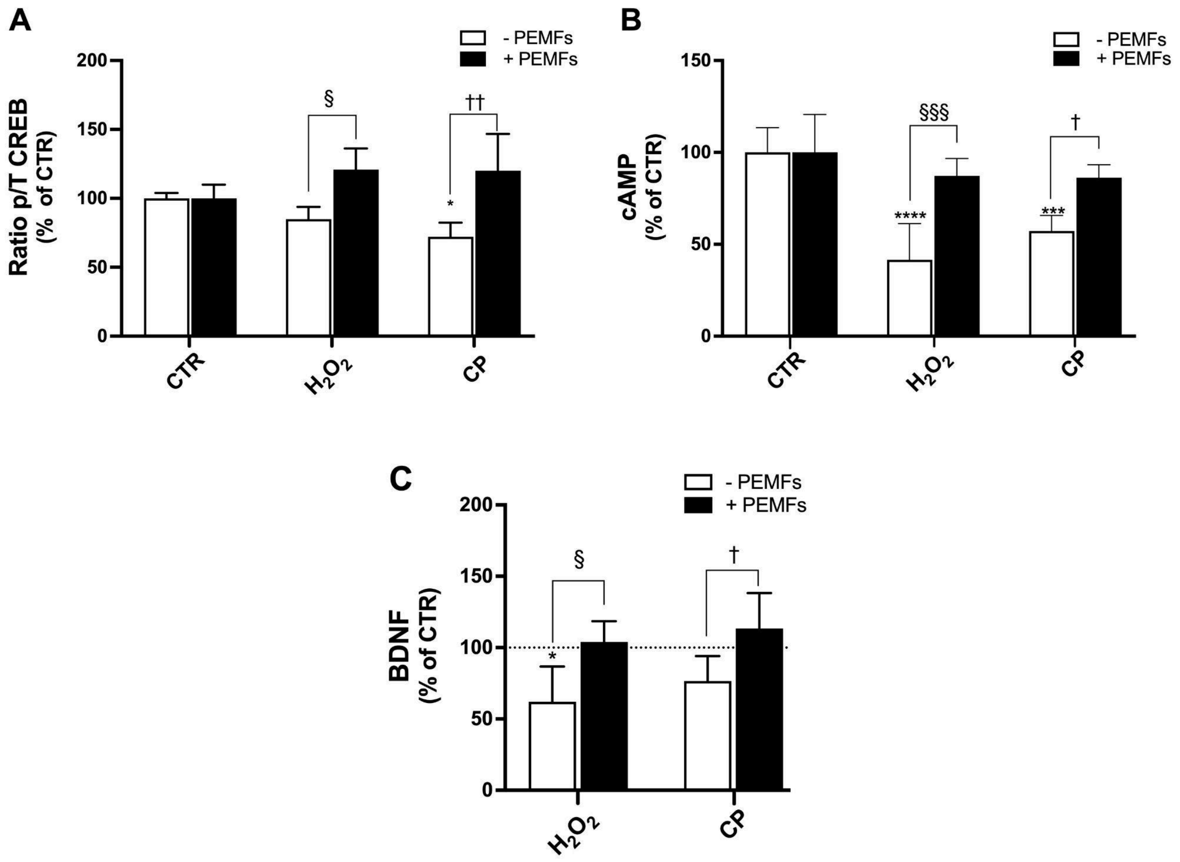

Figure S8. PEMFs' effects on CREB, cAMP and BDNF in PC12 cells. A. Total (T) and phosphorylated (p) forms of CREB after 1 mM H_2O_2 or 20 μM CP treatment of cells for 20 min. Results are presented as mean ± SD values of at least four independent experiments performed in duplicate (*p<0.05 vs control (CTR) without PEMFs; §p<0.05, vs H_2O_2-treated cells; ††p<0.01 vs CP-treated cells). **B**. cAMP production after 1 mM H_2O_2 and 20 μM CP cell treatment. Results are presented as mean ± SD values of at least three independent experiments performed in duplicate (****p<0.0001 and ***p<0.001 vs CTR without PEMFs, respectively; §§§p<0.001 vs H_2O_2-injured cells; †p<0.05 vs CP-injured cells). **C**. Extracellular BDNF levels after 200 μM H_2O_2 or 20 μM CP treatment for 24h. Results are presented as mean ± SD values of at least three independent experiments performed in duplicate (*p<0.05 vs CTR without PEMFs; §p<0.05 vs H_2O_2-injured cells; †p<0.05 vs CP-injured cells). Statistical analysis has been performed by one-way ANOVA and Sidak's multiple comparison test. cAMP, cyclic adenosine monophosphate; CREB, cAMP response element-binding protein; BDNF, brain-derived neurotrophic factor.

SUPPLEMENTARY FIGURE S9

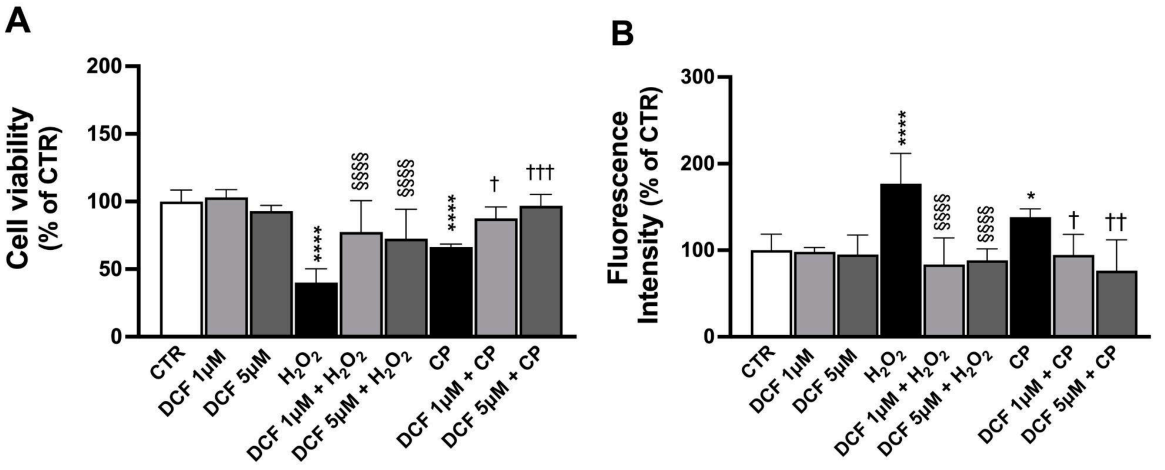

Figure S9. Diclofenac (DCF) effect in injured PC12 cells. Cell viability and ROS production were studied in cells pretreated for 30 min with 1 μM or 5 μM DCF and then treated with 1 mM H_2O_2 or 20 μM CP for 24h, through MTS (A) and H_2DCFDA (B) assays. **A.** Results are presented as mean ± SD values of at least four independent experiments performed in duplicate (****$p<0.0001$ vs control, CTR; $^{§§§§}p<0.0001$ vs H_2O_2-injured cells; $^{†}p<0.05$ and $^{†††}p<0.001$ vs CP-injured cells). **B.** The graph shows mean ± SD values from three independent experiments performed in duplicate (****$p<0.0001$ and *$p<0.05$ vs CTR; $^{§§§§}p<0.0001$ vs H_2O_2-injured cells; $^{†}p<0.05$ and $^{††}p<0.01$ vs CP-injured cells). Statistical analysis has been performed by one-way ANOVA and Sidak's multiple comparison test. MTS, 3-(4,5-dimethylthiazol-2-yl)-5-(3-carboxymethoxyphenyl)-2-(4-sulfophenyl)- 2H-tetrazolium.
