# Supplementary figures and images for: PEMFs Restore Mitochondrial and CREB/BDNF Signaling in Oxidatively Stressed PC12 Cells Targeting Neurodegeneration
Source: Int J Mol Sci. 2025 Jul 5;26(13):6495. doi: 10.3390/ijms26136495 (PMC12250253; doi:10.3390/ijms26136495)

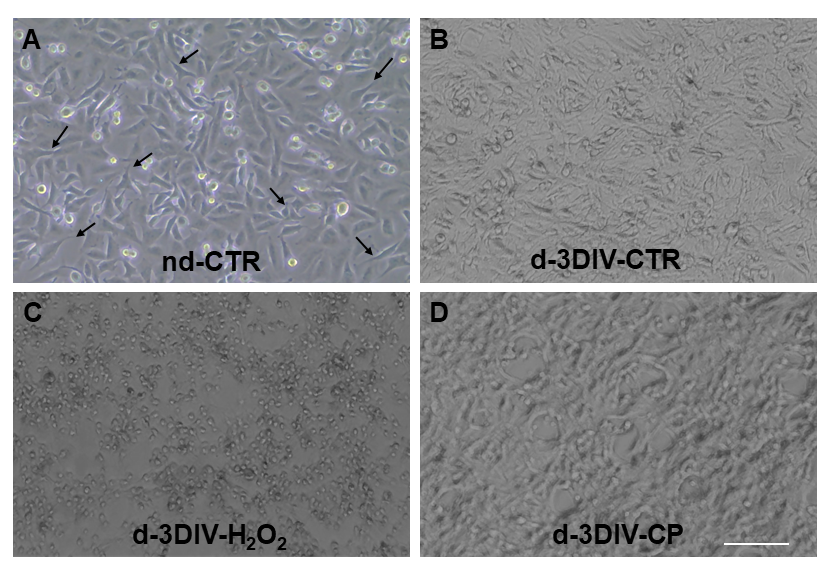

Supplement: Supplementary file 1 [file ijms-26-06495-s001.zip › Supplementary Figure S1.tif]

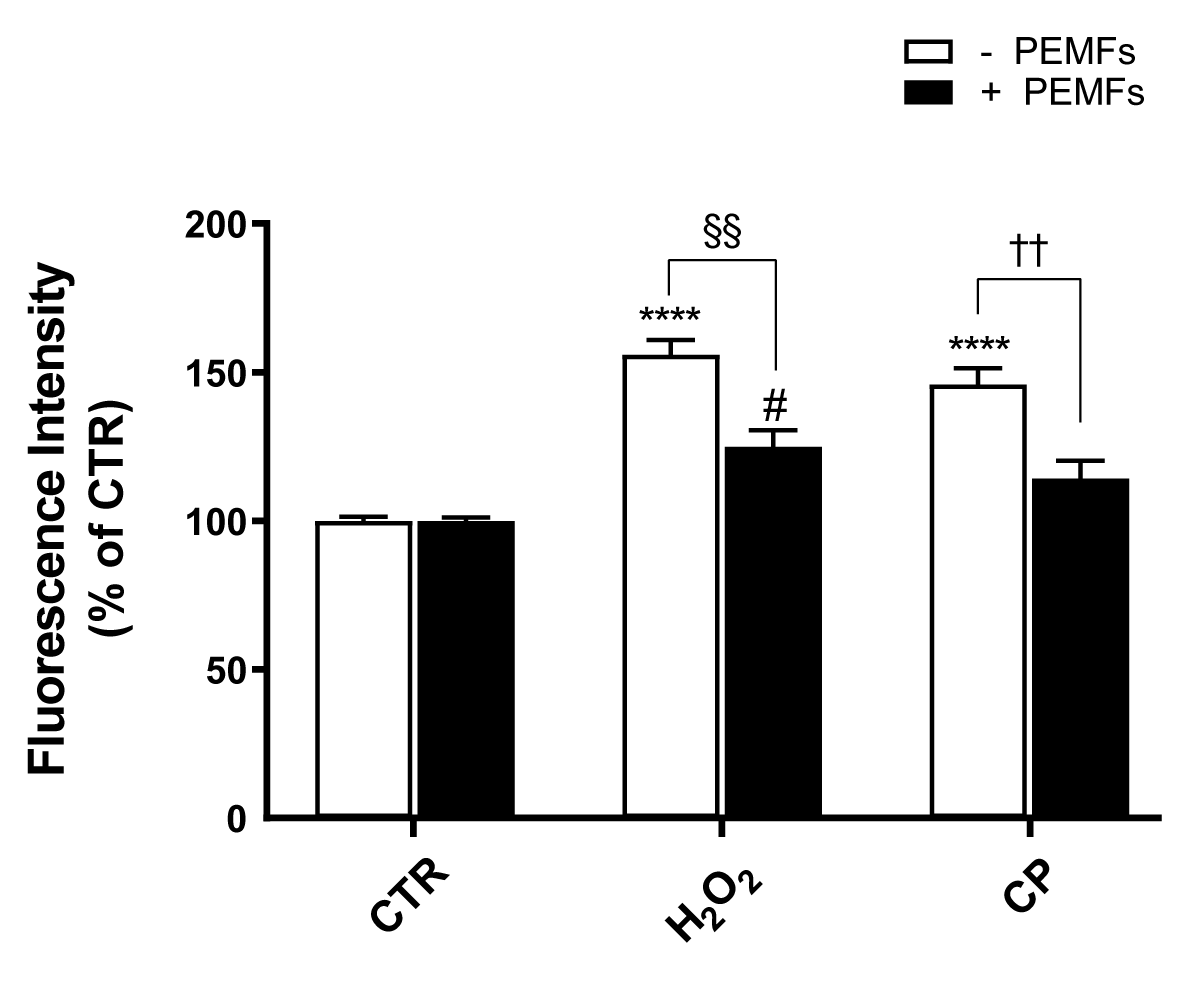

Supplement: Supplementary file 1 [file ijms-26-06495-s001.zip › Supplementary Figure S2.tif]
